# MSC-derived exosomes ameliorate erectile dysfunction by alleviation of corpus cavernosum smooth muscle apoptosis in a rat model of cavernous nerve injury

**DOI:** 10.1186/s13287-018-1003-1

**Published:** 2018-09-26

**Authors:** Xi Ouyang, Xiaoyan Han, Zehong Chen, Jiafeng Fang, Xuna Huang, Hongbo Wei

**Affiliations:** 1 Department of Gastrointestinal Surgery, The Third Affiliated Hospital of Sun Yat-sen University, Tianhe Road 600, Guangzhou, 510630 China; 2 Central Laboratory, The Third Affiliated Hospital of Sun Yat-sen University, Tianhe Road 600, Guangzhou, 510630 China

**Keywords:** Mesenchymal stem cells, Exosomes, Cavernous nerve injury, Erectile dysfunction, Apoptosis

## Abstract

**Background:**

This study investigated the therapeutic effects of MSC-derived exosomes (MSC-Exos) on erectile function in a rat model of cavernous nerve injury (CNI).

**Methods:**

MSCs were isolated from rat bone marrow and exosomes were isolated from the supernatants by ultracentrifugation. The tissue explant adherent method was used to isolate and culture corpus cavernosum smooth muscle cells (CCSMCs). MSCs and CCSMCs were identified by flow cytometry, in vitro differentiation or immunofluorescence staining. Thirty-two 10-week-old male Sprague Dawley (SD) rats were divided into four groups: a sham operation group and bilateral CNI groups that received intracavernosal (IC) injection of either PBS, MSCs or MSC-Exos. Four weeks after CNI and treatment, the erectile function of the rats was measured by electrically stimulating the cavernous nerve. The penile tissues were harvested for blinded histologic analysis and western blotting. H_2_O_2_ was used to induce apoptosis in the CCSMCs, and a flow cytometer was used to measure the cell viability of the CCSMCs treated with or without exosomes in vitro.

**Results:**

Recovery of erectile function was observed in the MSC-Exos group. The MSC-Exos treatment significantly enhanced smooth muscle content and neuronal nitric oxide synthase in the corpus cavernosum. The ratio of smooth muscle to collagen in the corpus cavernosum was significantly improved in the MSC-Exos treatment group compared to the PBS vehicle group. WB confirmed these biological changes. Cell viability of the CCSMCs was increased in the MSC-Exos-treated groups, and caspase-3 expression was decreased after the MSC-Exos treatment in vivo and in vitro.

**Conclusions:**

Exosomes isolated from MSCs culture supernatants by ultracentrifugation could ameliorate CNI-induced ED in rats by inhibiting apoptosis in CCSMCs, with similar potency to that observed in the MSCs-treated group. Therefore, this cell-free therapy has great potential for application in the treatment of CNI-induced ED for replacing cell therapy.

**Graphical abstract:**

MSC-derived exosomes ameliorate erectile dysfunction in a rat model of cavernous nerve injury
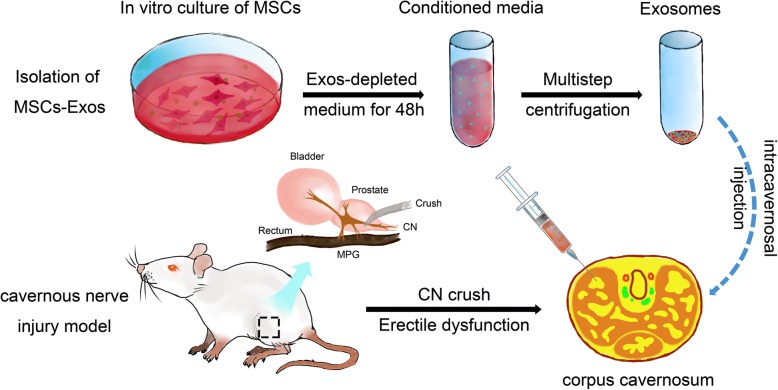

## Background

Pelvic surgeries for prostate and colorectal cancer commonly result in a high incidence of erectile dysfunction (ED) due to damage of the cavernous nerve (CN) [1, 2]. Despite technical and anatomical advances, especially with nerve-sparing techniques, that have been applied to avoid sexual dysfunction, ED remains a major complication of these surgeries [3]. Many studies have suggested that increased apoptosis of corpus cavernosum smooth muscle cells (CCSMCs) is a common etiology in cavernous nerve injury (CNI)-induced ED and CNI-induced damage to corporal smooth muscle cells is always irreversible [4–8]. Penile rehabilitation with phosphodiesterase type 5 inhibitors (PDE5Is) is currently the most commonly used treatment after pelvic surgeries [9]. Several preclinical studies using rodent models have shown that PDE5Is treatment can promote penile rehabilitation through reduced penile apoptosis [10, 11]. However, more recently, many large randomized, controlled clinical trials have suggested that regular dosing of a PDE5Is following pelvic surgeries cannot prevent the deterioration of erectile function [9, 12, 13]. Therefore, there is a great need to develop more effective novel strategies aimed at decreasing apoptosis of the CCSMCs to treat CNI-induced ED.

Recent research approaches for CNI-induced ED include the use of gene therapy and stem cell-based therapies. We also found that intracavernous (IC) injection of mesenchymal stem cells (MSCs) significantly improved erectile function in a rat model of CNI-induced ED [14]. Despite the unknown mechanism of this transplantation approach, it has been increasingly observed that the effect of tissue repair of MSCs is not by engraftment in tissues or differentiation into specialized cell types but by secreting abundant bioactive substances [15, 16]. However, it is now clear that, in addition to secreted soluble factors, MSCs are able to secrete proteins, lipids, and nucleic acids in extracellular vesicles (EVs) as a method of influencing their host environment [17–19].

Among the many types of EVs, the exosomes, are generated inside multivesicular endosomes or multivesicular bodies and have a size of 40 to 150 nm in diameter [20]. Recent studies indicated that MSC-derived exosomes (MSC-Exos) are efficacious in animal models of stroke [21], hind-limb ischemia [22], cutaneous wounds [23], and kidney diseases [24]. To our knowledge, whether MSC-Exos can be exploited following transplantation to recover CNI-induced ED remains largely unknown. In this study, we aimed to determine the efficacy of IC injection of MSC-Exos to treat CNI-induced ED according to the hypothesis that the MSC-Exos might exert their beneficial effects on alleviating ED through decreasing the apoptosis of CCSMCs. The results of the present study may suggest a novel therapeutic strategy for CNI-induced ED.

## Methods

### Animal models and experimental design

Ten-week-old male Sprague-Dawley rats were obtained from the Guangdong Medical Laboratory Animal Center (Guangzhou, China). The animals were maintained on a 12-h light-dark cycle and had access to water ad libitum at the Center for Experimental Animals at the Third Affiliated Hospital of Sun Yat-sen University. The care and treatment were approved by the ethics committee of the Institutional Animal Care and Use Subcommittee of the Third Affiliated Hospital of Sun Yat-sen University.

Thirty-two male SD rats (10-weeks-old) were assigned to four groups (*n* = 8 per group) and subjected to CNI or sham surgery and then treated with IC injection of phosphate-buffered saline (PBS), MSCs, or MSC-Exos. At 4 weeks after surgery, erectile function was measured for all rats. Then, the penile tissues were harvested for blinded histologic analysis and western blotting.

### Isolation and characterization of rat bone marrow-derived MSCs

Rat bone marrow MSCs were isolated from 4-week-old Sprague-Dawley rat femurs as previous published [25]. The cells were cultured in Dulbecco’s modified Eagle’s medium supplemented with 10% fetal bovine serum (FBS) at 37 °C in 5% CO_2_. All non-adherent cells were removed, and the medium was changed every 3 days. The following antibodies were used for verification of surface marker: CD29, CD44, CD90, CD11b, CD34, and CD45 (eBioscience Inc., San Diego, CA, USA). The culture-grown MSCs were tested for their ability to differentiate into adipocytes and osteoblasts as described previously [26]. MSCs were cultured in the following medium types: (1) adipogenic differentiation medium (Dulbecco’s modified Eagle medium [DMEM] with 1 g/ml glucose, DMEM-LG) containing 10% FBS, 50 μg/ml of ascorbate-1 phosphate, 0.1 μmol/L dexamethasone and 50 μg/ml indomethacin; (2) osteogenic differentiation medium (DMEM-LG containing 10% FBS, 50 μg/ml ascorbate-2 phosphate, 10–2 μmol/L dexamethasone, and 10 mmol/L β-glycerophosphate); (3) chondrogenic differentiation medium (DMEM-LG containing 1% FBS, 50 μg/ml ascorbate-2 phosphate, 6.25 μg/mL insulin transferrin selenium, and 10 ng/mL tumor growth factor beta [TGF-β]). The medium was changed every 3 days. Adipocytes were identified by oil-red O staining, osteoblasts by von Kossa staining, and chondrogenesis by Alcian blue staining. For all experiments described, the cells were used between passages 3–5.

### Isolation and characterization of MSC-derived exosomes (MSC-Exos)

For the preparation of exosomes-depleted FBS, FBS was ultracentrifuged at 4 °C at 120,000×*g* for 14 h using a SW28 swinging-bucket rotor in an ultracentrifuge (Optima-90 K, Beckman Coulter, Brea, CA, USA). The supernatant was filtered using a 0.22-μm syringe-filter and stored at 4 °C. As described in the previously published protocol [27], conventional culture medium was replaced with exosomes-depleted culture medium when the cells reached 80% confluence, and the MSCs were cultured for an additional 48 h. Then, the medium was collected, and exosomes were isolated through multistep centrifugation. Media was centrifuged at 300 g for 10 min, 2000 g for 20 min, and 10,000 g for 30 min to eliminate dead cells and debris. Then, the supernatant was ultracentrifuged at 100,000 *g* for 90 min, and the pellet was washed with PBS before centrifugation at 100,000 *g* for 90 min (Optima- 90 K, Beckman Coulter).

The pellets were resuspended in PBS. Exosomes size distribution analysis was done using the qNano® system (Izon Science, Oxford, UK) according to the manufacturer’s instructions. The total protein concentration in the exosomes was quantitated using a Micro Bicinchoninic Acid Protein Assay Kit (Pierce, Rockford, IL, USA), according to the manufacturer’s recommended protocol. Protein levels of CD63 (ProteinTech, Chicago, IL, USA, 25682–1-AP, 1:1000), TSG101 (Abcam, Cambridge, UK, ab125011, 1:2000), and Flotillin-1 (Abcam, Cambridge, UK, ab133497, 1:10000) were determined using western blot. The morphology and ultrastructure of exosomes were analyzed using transmission electron microscopy.

### Isolation and characterization of corpus cavernosum smooth muscle cells (CCSMCs)

Explant cell cultures were prepared following the protocols described by other authors [27]. Briefly, the skin overlying the penis was incised and bilateral penile crura were exposed by removing part of the ischiocavernosus muscle and fascia. Then, the cavernosal tissue was washed in PBS and cut into 1–2 mm^3^ pieces. Segments were placed on 100 mm cell culture dishes (Corning, Corning, NY, USA) with a minimal volume of DMEM, supplemented with 20% FBS, 100 U/ml penicillin, and 100 mg/ml streptomycin and cultured at 37 °C in a humidified atmosphere of 95% air and 5% CO_2_. After the explants attached to the substrate, more DMEM containing 10% FBS was added, and tissue segments that had detached from the dishes were removed. After cells migrated out from the explants, the explants were removed, and cells were allowed to achieve confluence. Immunofluorescence was performed for cell identification with an anti-calponin antibody (Santa Cruz Biotechnology, Dallas, TX, USA, SC58707, 1: 100) from passage 3 cells.

### MSC-Exos uptake in vivo and in vitro

For the evaluation of exosomes uptake in the cavernosum after treatment, exosomes were labeled with a green fluorescent dye (PKH67, Sigma-Aldrich, St. Louis, MO, USA) as previously described [28]. Labeled exosomes were injected into the cavernosum immediately after bilateral CNI. Frozen sections were prepared, and 4′,6-diamidino-2-phenylindole (DAPI; 0.5 μg/mL; Invitrogen, Carlsbad, USA) staining was analyzed by immunofluorescence staining at 24 h. To determine MSC-Exos uptake by CCSMCs, CCSMCs were grown in the wells of 24-well plates and then incubated with labeled exosomes (10 μg/ml) at 37 °C for 4 h, 8 h and 16 h. The cells were then washed three times with PBS and fixed with 4% PFA for 10 min. After washing with PBS, nuclei were stained with DAPI, and fluorescence microscopy was used to detect the green signals in CCSMCs.

### Apoptosis induction and cell viability assay

To induced apoptosis in CCSMCs, H_2_O_2_ was added to the culture medium at a final concentration of 200 μΜ. To measure cell viability, cells were seeded in six-well plates at a density of 2 × 10^5^ cells/cm^2^ and incubated for 24 h. After washing, cells were incubated with exosomes-depleted media containing FBS with or without exosomes at 10 μg/ml or 20 μg/ml for 6 h and then treated with H_2_O_2_ for 18 h to induce apoptosis. Then, the cells were collected, washed twice with PBS, and stained with Annexin-V-propidium iodide double staining. Cell viability was measured using a flow cytometer (BD FACSCanto™, Becton, Dickinson and Company, Franklin Lakes, NJ, USA).

### CNI and sham surgery

To produce bilateral CNI, the rats were first weighed and anesthetized with 2.5–3% isoflurane. The nerve crush site was 2–5 mm distal to the major pelvic ganglion (MPG) and injury was induced as previously described [14]. The sham surgery was performed in exactly the same way except no nerves were crushed.

### IC injection

For IC injections, the prepuce was rolled up to expose the penis, allowing injection to the lateral aspect of the penis. The needle was inserted 3–4 mm. All rats received an IC injection of PBS 0.1 mL (PBS group), MSCs (1.5 × 10^6^ cells in PBS 0.1 mL; MSCs group), or MSC-Exos (100 μg in PBS 0.1 mL; MSC-Exos group).

### Erectile function evaluation

Both intracavernous pressure (ICP) and mean arterial blood pressure (MAP) were recorded continuously as previously described [29, 30]. Under anesthesia, a midline incision from the neck to the upper thorax was made to expose the right carotid artery. Then, a heparinized 24-gauge silastic cannula was inserted to measure MAP. The MPG and cavernous nerve were exposed via midline incision. The corpora cavernosum of the penis was cannulated with a heparinized (250 U/ml) 25-gauge butterfly needle via insertion at the crura. The cannula was connected to a BL-420 s Biological Functional System (Chengdu Taimeng Technology Ltd., Chengdu, China) for continuous assessment and recording of ICP. The stimulus parameters were 1.5 mA, 20 Hz, pulse width 0.2 ms and duration 50 s [30]. The maximum increase in ICP for three stimuli per side for each animal was selected for statistical analysis of mean ICP. The penis, MPG, and distal cavernous nerve were then harvested for histologic analysis and western blotting.

### Histologic analysis

Penile midshaft tissues were harvested and immediately fixed for immunofluorescence staining, which was performed as previously described [14]. The penile segment sections were incubated with primary antibodies to anti-neuronal nitric oxide synthase (Cell Signaling Technology Co., Ltd., Danvers, MA, USA, #4231, 1:200) and anti-smooth muscle actin (Cell Signaling Technology Co., Ltd., #48938, 1:500). Secondary antibodies included DyLight 488 and 556-conjugated antibodies (Invitrogen, Waltham, MA, USA, 1:500). Nuclei were stained with DAPI (0.5 μg/mL; Invitrogen, Carlsbad, CA, USA). Signals were visualized, and digital images were obtained with a confocal laser scanning microscope (Zeiss LSM 710, Oberkochen, Germany). Masson trichrome staining was used to quantify the ratio between the smooth muscle and collagen within the corpus cavernosum as previously described [30]. Image analysis was performed by using Image-Pro Plus 5.1 (Media Cybernetics, Silver Spring, MD, USA).

### TUNEL staining

Apoptotic cells in the penile midshaft tissues were identified and quantified by using an in situ cell apoptosis kit (Beyotime Biotechnology, Shanghai, China) according to the manufacturer’s protocols. Images were captured, and apoptotic cell nuclei were characterized by red fluorescence.

### Western blotting

For western blot analysis, tissues and cells were lysed with RIPA buffer containing protease inhibitor cocktail, and the protein concentrations of tissue lysates and cell lysates were determined by BCA assay. Samples containing 20 μg of protein were subjected to sodium dodecyl sulfate polyacrylamide gel electrophoresis and transferred to a polyvinylidene fluoride membrane. The membrane was blocked with 5% skim milk and incubated at 4 °C overnight with primary antibodies against nNOS, anti-smooth muscle actin, caspase-3 (Cell Signaling Technology Co., Ltd., #9662, 1:1000) or b-actin (Cell Signaling Technology Co., Ltd., #4970,1:1000). After hybridization of secondary antibodies, the resulting images were analyzed using Tanon 5200 (Tanon Science & Technology Co., Ltd., Shanghai, China) to determine the integrated density for each protein band.

### Statistical analyses

The results were analyzed using GraphPad Prism (v.5) software (GraphPad Software, La Jolla, CA, USA) and expressed as the mean ± standard deviation. Multiple groups were compared using one-way analysis of variance (ANOVA) followed by the Tukey-Kramer test for post hoc comparisons using SPSS 16.0 software (SPSS Inc., Chicago, IL, USA). *P* < 0.05 was considered statistically significant.

## Results

### Isolation and characterization of MSCs

**Fig. 1:**
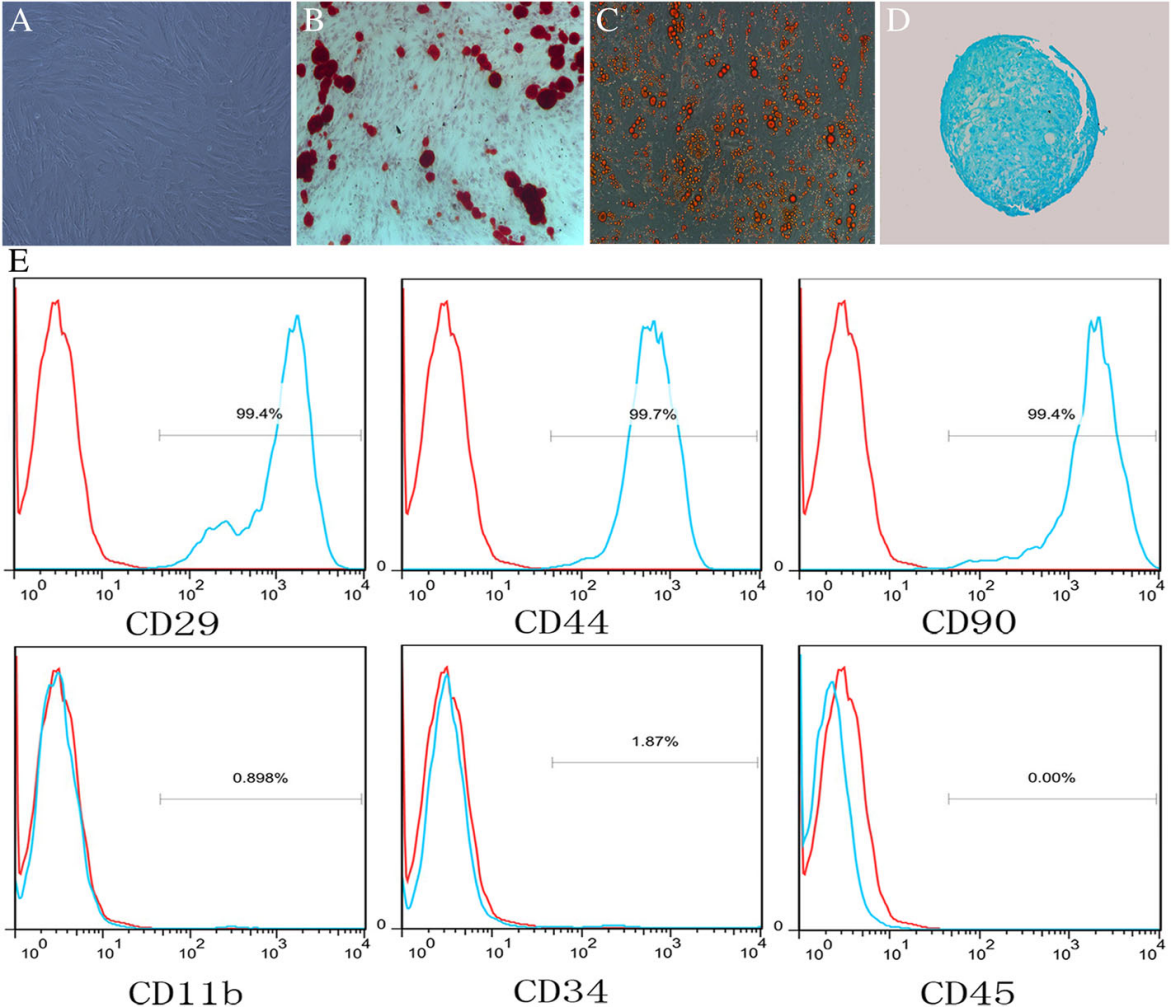
Isolation and characterization of MSCs. **a** The morphology of MSCs. **b** After induction, the cells possessed the typical phenotypes of osteocytes (*stained with Alizarin Red S*). **c** After induction, the cells possessed the typical phenotypes of adipocytes (*stained with Oil Red O*). **d** After induction, the cells possessed the typical phenotypes of chondrogenesis (*stained with Alcian blue staining*). **e** Differentiated cells express the MSCs markers, CD29, CD40, and CD90, but do not express the hematopoietic or endothelial markers, CD11b, CD34, and CD45

Primary MSCs were isolated from the femurs of 4-week-old Sprague-Dawley rats and cultured in vitro. MSCs in this study showed typical spindle, fibroblast-like morphology, and the colonies were arranged in a circular whirlpool-like fashion (Fig. 1a). To characterize the MSCs used in this study, we first assessed the MSCs in vitro potential to differentiate into adipocytes and osteocytes. The results showed that the cells were stained positive with Alizarin Red after osteogenic induction **(**Fig. 1b), stained positive with Oil Red O after adipogenic induction (Fig. 1c) and stained positive with Alcian blue after chondrogenic induction (Fig. 1d). We further analyzed the expression of cell surface antigens, as shown in Fig. 1e, the cells expressed markers of MSCs, including CD29, CD44, and CD90, but not the hematopoietic or endothelial markers, CD11b, CD34, and CD45.

### Characterization of MSC-Exos

**Fig. 2:**
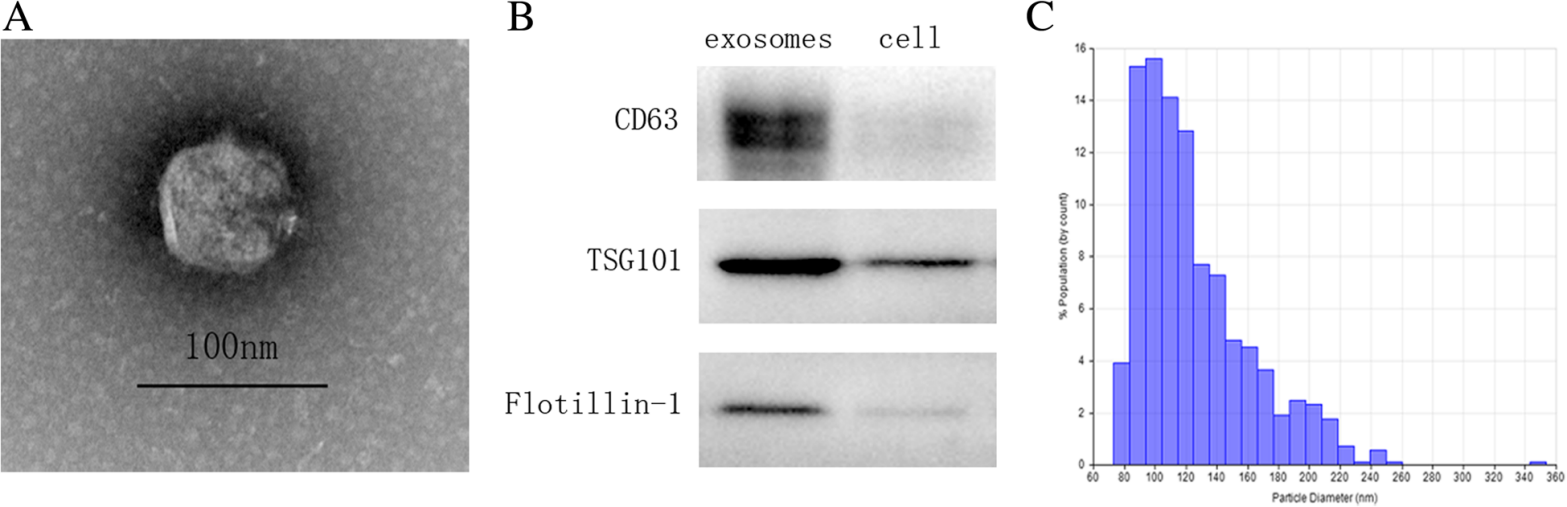
Isolation and characterization of MSC-Exos. **a** Representative transmission electron micrographs of MSC-Exos with a cup-shaped morphology. Scale bar = 100 nm **b** Western blot results indicating positive expression for the CD63, TSG101, and Flotillin-1 proteins in the exosomes derived from MSCs. **c**. Particle size distribution of MSC-Exos measured by qNano analysis

Transmission electron microscopy (TEM), western blot analysis, and qNano analysis were performed to identify the purified nanoparticles derived from MSCs. TEM analysis revealed that MSC-Exos isolated from the supernatants exhibited a cup-shaped morphology (Fig. 2a), similar to previously described exosomes [20]. The expression of exosomal markers CD63, TSG101, and Flotillin-1 were further quantified by western blot analysis (Fig. 2b). qNano measurement indicated that the diameters of typical rounded particles mainly ranged from 70 nm to 140 nm (Fig. 2c), which was consistent with the previously reported exosomes size distributions [20].

### MSC-Exos treatment improves erectile function after CNI

**Fig. 3:**
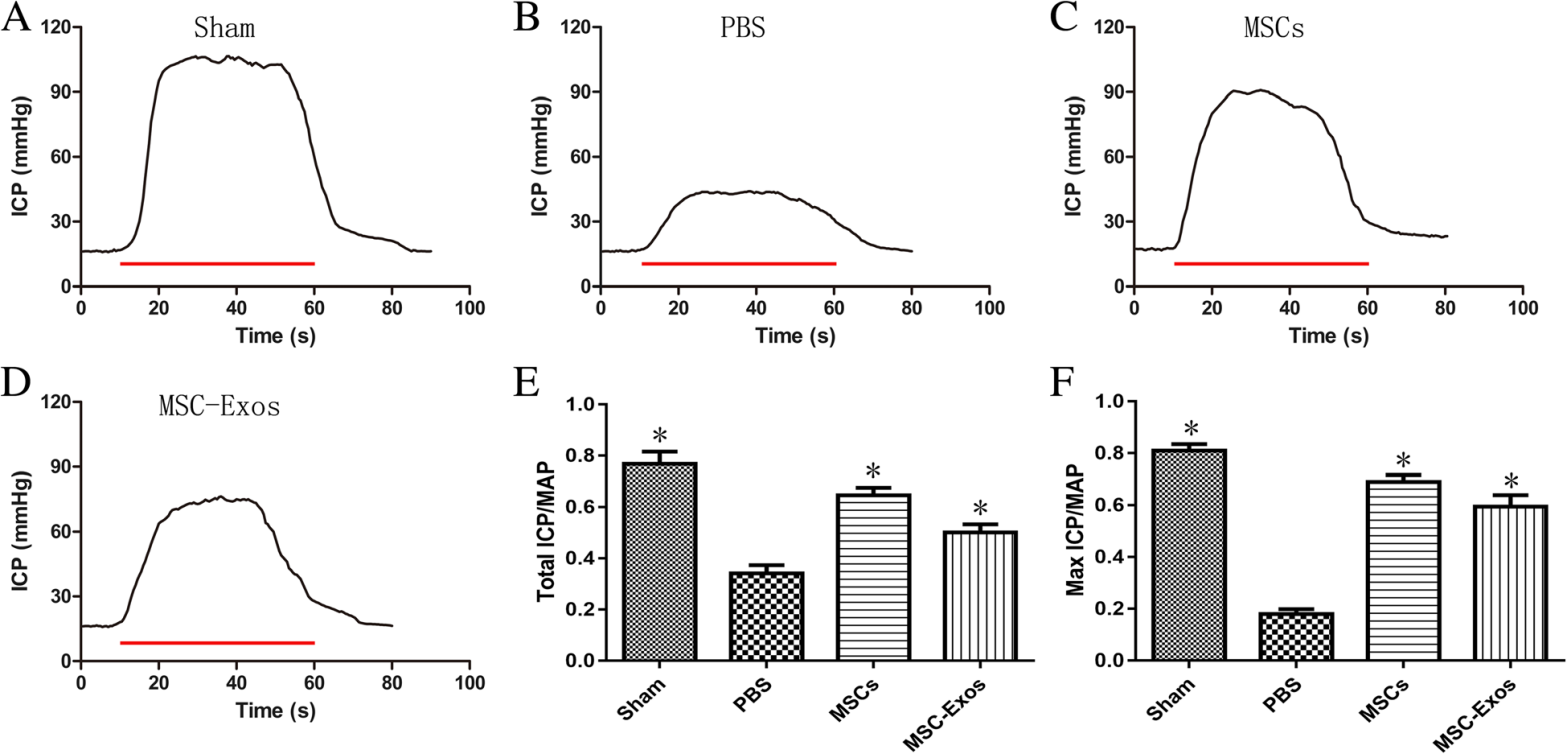
Intracavernous pressure (ICP) during cavernous nerve (CN) electrostimulation at 4 weeks after surgery. **a-d** Representative ICP responses for the sham group, CNI rats stimulated 4 weeks after IC injection of PBS, MSCs, and MSC-Exos. The *colored bar* denotes the 50 s CN electrical stimulation. **e, f** The ratios of the total ICP (area under the curve) and maximal ICP to MAP are recorded. Each bar depicts the mean ± standard deviation from *n* = 8 animals per group. *p < 0.01 compared with the PBS vehicle group

The effects of MSC-Exos treatment on the recovery of erectile function are illustrated in Fig. 3. The maximal ICP (mICP) and total ICP (tICP, area under the curve) were both determined for data analysis. The sham group exhibited normal ICP curves and high tICP/MAP ratios and mICP/MAP ratios, whereas CNI consistently resulted in ED. The tICP/MAP ratios and mICP/MAP ratios were lower for the PBS group (0.34 ± 0.03, 0.18 ± 0.02) than for the sham group (0.77 ± 0.05, 0.81 ± 0.03). Recovery of erectile function to varying degrees was observed in the MSCs and MSC-Exos treatment groups as reflected by significantly higher tICP/MAP ratios and mICP/MAP ratios in response to cavernous nerve electrical stimulus (MSCs, 0.65 ± 0.03, 0.69 ± 0.03; MSC-Exos, 0.50 ± 0.03, 0.60 ± 0.04) compared to the PBS group (*p* < 0.01).

### Transplantation of MSC-Exos increase penile nNOS expression and alleviate cell apoptosis

**Fig. 4:**
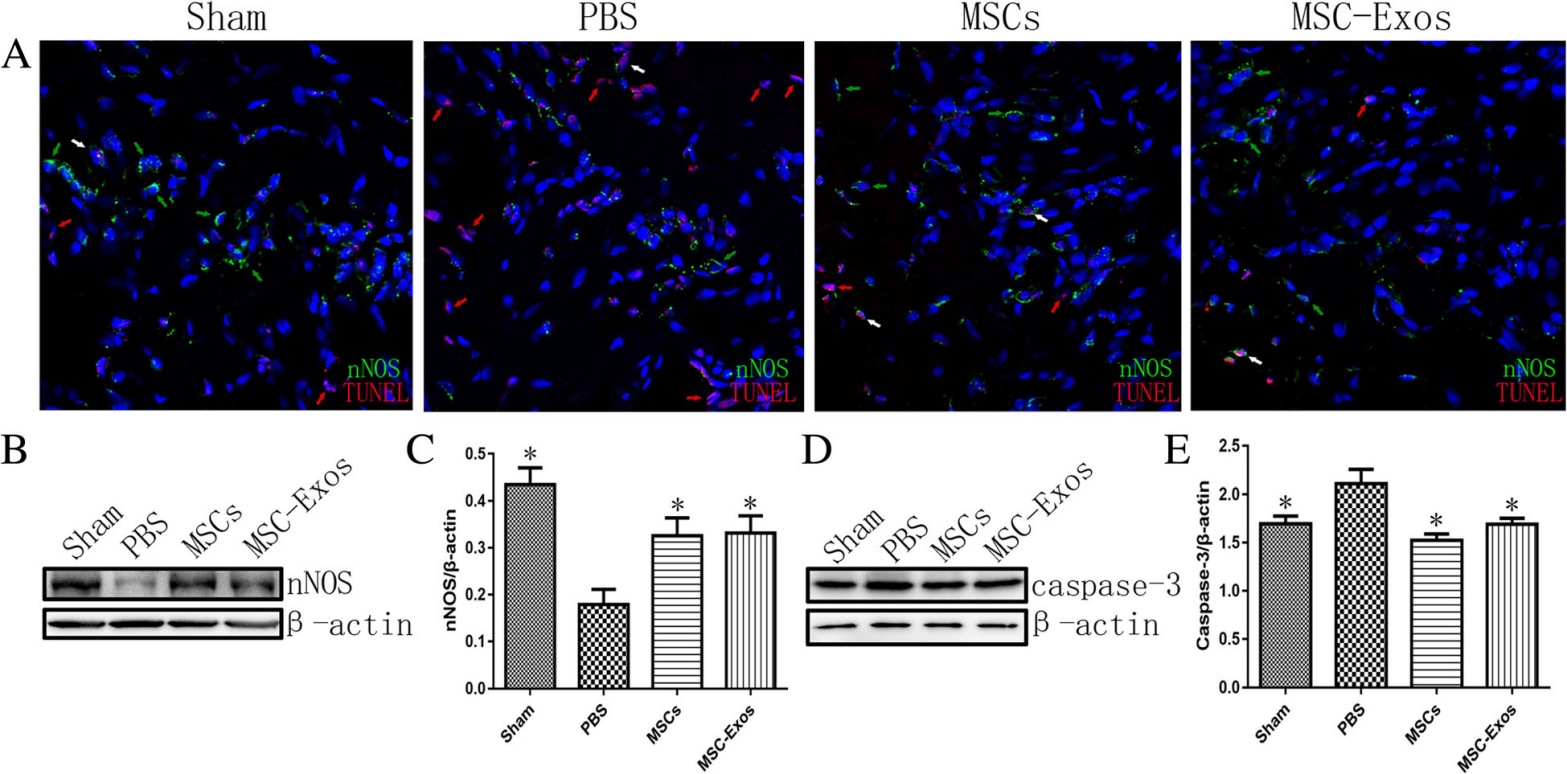
Neuronal nitric oxide synthase (nNOS) and caspase-3 expression in the cavernosum. **a** Representative immunofluorescence staining of nNOS (*green*) and TUNEL (*red*) in a penile midshaft specimen, 4 weeks after CNI and treatment (nNOS positive cells—*green arrowheads*, TUNEL positive nuclei—*red arrowheads*, nNOS and TUNEL both positive cells—*white arrowheads*). **b** Representative images of western blots for nNOS in cavernosum from each group. Original magnification, ×400. DAPI = 4′,6-diamidino-2-phenylindole. **c** Data are presented as the relative density of nNOS compared with that of β-actin. **d** Representative images of western blots for caspase-3 in cavernosum from each group. **e** Data are presented as the relative density of caspase-3 compared with that of β-actin. Each bar depicts the means ± standard deviation from n = 8 animals per group. *p < 0.05 compared with the PBS vehicle group

The expression of nNOS in the corpus cavernosum was detected by immunofluorescent staining 4 weeks after injection with MSCs or MSC-Exos. The data revealed that nNOS expression was significantly higher in the sham (0.43 ± 0.04), MSCs (0.33 ± 0.04), and MSC-Exos (0.33 ± 0.10) groups than in the PBS group (0.18 ± 0.03; *p* < 0.01) (Fig. 4a, b, c). TUNEL assays were applied to assess cell apoptosis in the corpus cavernosum in vivo. In vivo TUNEL assays confirmed that MSC-Exos effectively protected cells from apoptosis after CNI. Additionally, MSC-Exos could alleviate the apoptosis of nNOS-positive cells, which also contributed to the recovery of erectile function (Fig. 4a). Western blots were performed to evaluate the expression of caspase-3 after 4 weeks of treatment with MSCs and MSC-Exos. The expression of caspase-3 significantly decreased in the MSCs (1.52 ± 0.07) and MSC-Exos (1.70 ± 0.06) groups compared to the PBS group (2.11 ± 0.15) (Fig. 4d, e).

### MSC-Exos treatment improves smooth muscle content and the ratio of smooth muscle to collagen in the corpus cavernosum

**Fig. 5:**
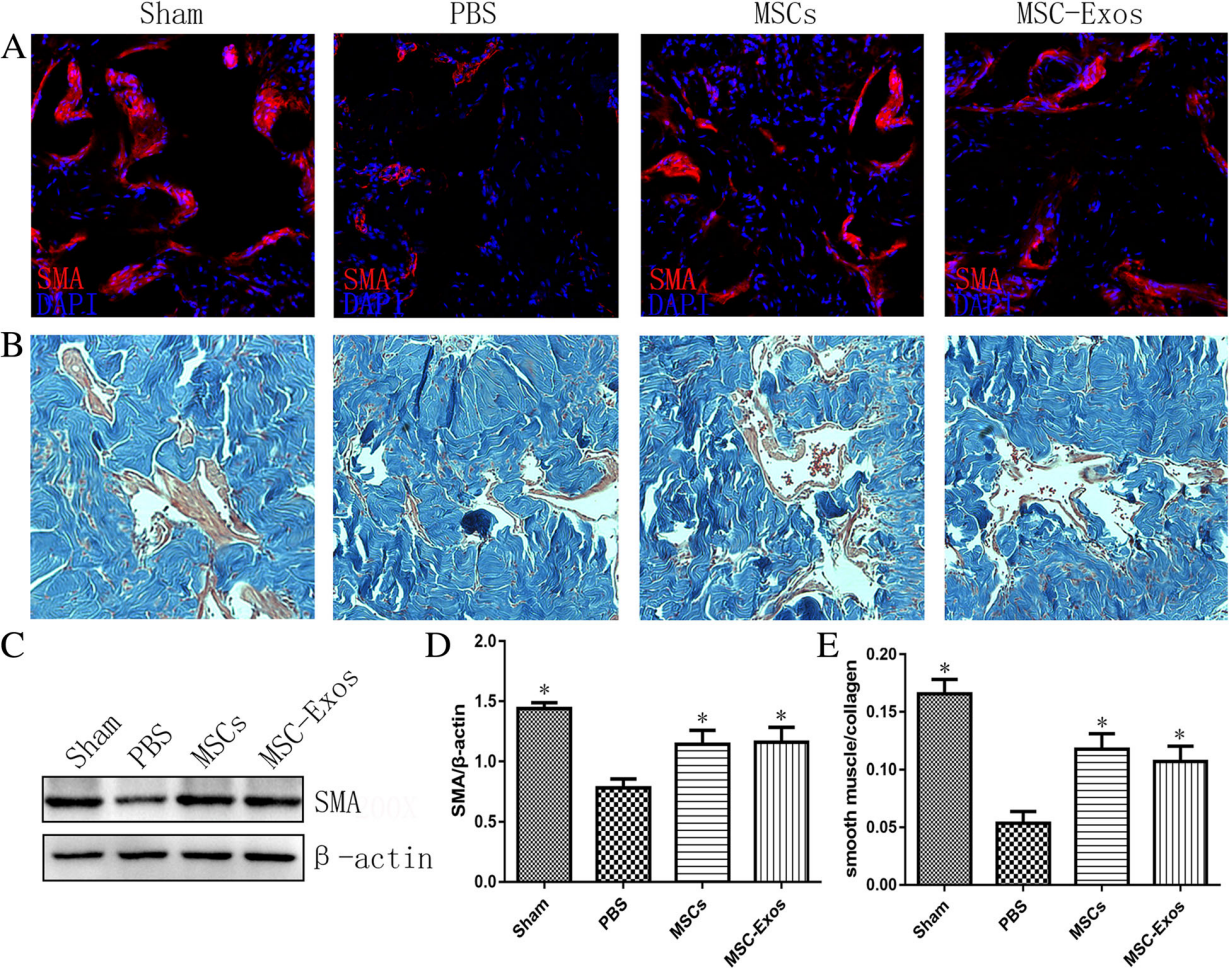
Transplantations of MSCs or MSC-Exos increase the smooth muscle contents of the corpus cavernosum. **a** The expressions of SMA in the corpus cavernosum were detected at 4 weeks in each group. Original magnification, ×200 DAPI = 4′,6-diamidino-2-phenylindole. **b** Representative images of Masson trichrome staining of actin and collagen in each group. Smooth muscle and connective tissue in the corpus cavernosum are stained *red* and *blue,* respectively. Original magnification, ×200. **c** Representative images of western blots for SMA cavernosum in each group. **d** Data are presented as the relative density of SMA compared with that of β-actin. The density was determined semiquantitatively using ImageJ. Each bar depicts the means ± standard deviations from n = 8 animals per group. *p < 0.05 compared with the PBS vehicle group. **e** Effect of MSCs or MSC-Exos treatment on the ratio of smooth muscle to collagen in the corpus cavernosum. Bars denote the mean densitometry ratio between smooth muscle content and collagen content per field (± standard error of the mean). *p < 0.05 compared with the PBS vehicle group

CNI caused corpus cavernosum smooth muscle atrophy, and computerized histomorphometric analysis showed a significant decrease in smooth muscle content in the corpus cavernosum of the PBS group compared to the sham group (Fig. 5a). The MSCs and MSC-Exos treatment groups exhibited partial but significant restoration of smooth muscle content after CNI, as shown by western blot analysis (Fig. 5c, d). The corpus cavernosum was evaluated for the smooth muscle/collagen ratios on slides stained with Masson’s trichrome (Fig. 5b). In the sham group, the smooth muscle/collagen ratio was 0.166 ± 0.013. In the penile tissue from rats from that received MSCs or MSC-Exos, the smooth muscle/collagen ratio (0.118 ± 0.013 or 0.107 ± 0.013) was almost fully preserved and significantly higher than in the PBS group (0.054 ± 0.010) (Fig. 5e, *p < 0.01).

### CCSMCs identification

**Fig. 6:**
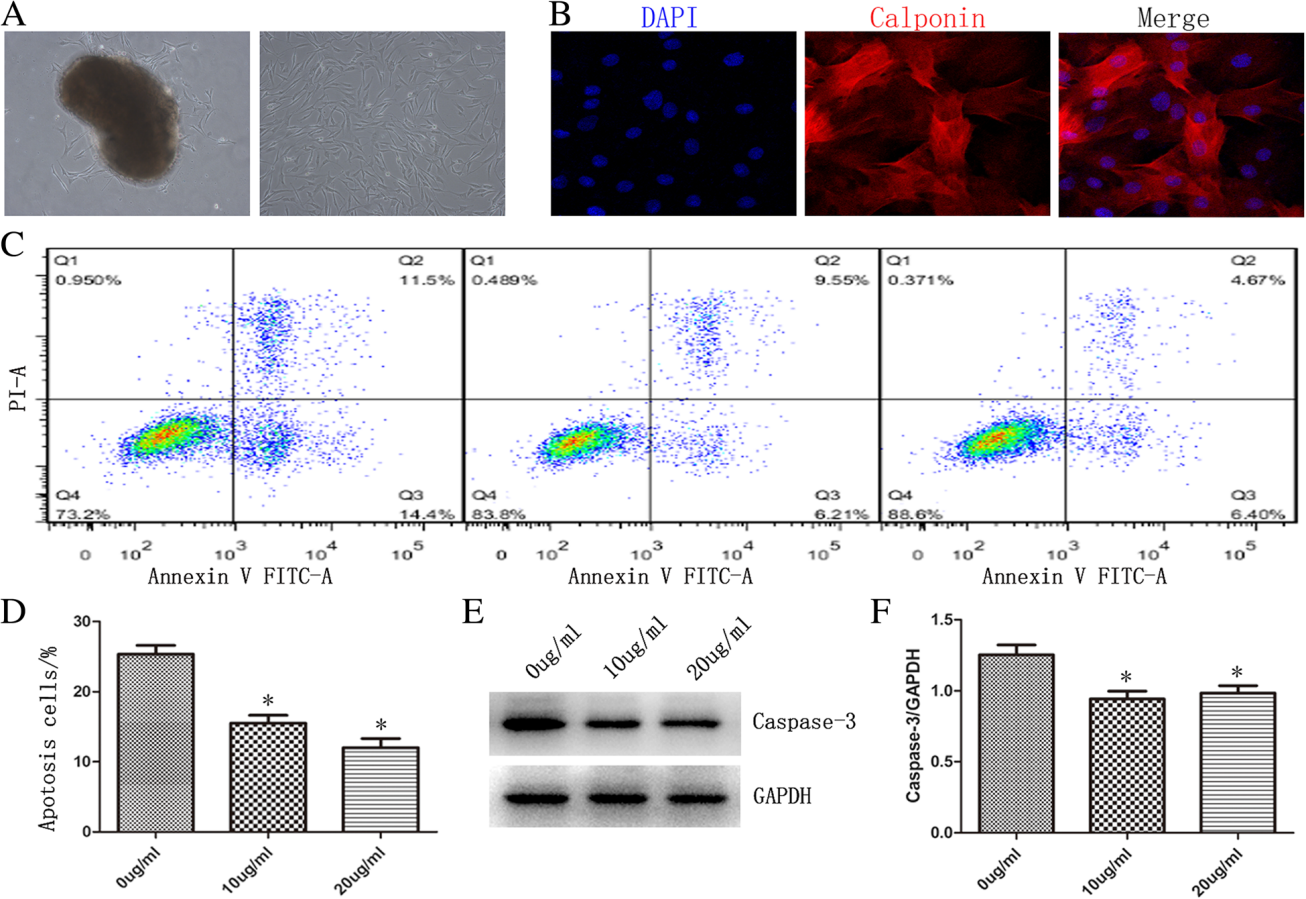
Effect of exosomes on CCSMC apoptosis. **a** The primary CCSMCs emerging from corporal tissue (*left*) and the passaged cells growing in a whirlpool-like pattern (*right*), at ×100 magnification. **b** Immunofluorescence with anti-calponin antibody for CCSMC identification, ×100 amplification. **c** Flow cytometric analysis of cell viabilities. Cells were cultured in the absence (0 μg/ml) or presence (10 μg/ml, 20 μg/ml) of MSC-Exos followed by H_2_O_2_ treatment. **d** The average apoptotic cell percentages are shown for cells cultured in the absence (0 μg/ml) or presence (10 μg/ml, 20 μg/ml) of MSC-Exos. All values are represented as the mean ± SD (*p < 0.05; *n* = 3). **e**. Western blot analysis of expression of caspase-3 in CCSMCs cultured in the absence (0 μg/ml) or presence (10 μg/ml, 20 μg/ml) of MSC-Exos followed by H_2_O_2_ treatment. **f** Data are presented as the relative density of caspase-3 compared with that of β-actin. All values are represented as the mean ± SD (*p < 0.05; n = 3)

Fig. 6 shows primary cells that have migrated out from the corpora tissues after 4–7 days and achieved confluence after 2–3 weeks. It also shows passaged cells growing in a whirlpool-like pattern (Fig. 6a). Over 95% of the cultured cells were identified by immunofluorescence (IF)-labeling of calponin (Fig. 6b), suggesting highly pure CCSMCs for the following experiments.

### Inhibition of CCSMCs apoptosis using exosomes

To assess the anti-apoptotic effects of MSC-Exos on CCSMCs, we analyzed the viability of exosomes-supplemented cells after H_2_O_2_ treatment by flow cytometry. CCSMCs were cultured in the absence or presence of exosomes for 6 h. Different concentrations of exosomes (10 μg/ml, 20 μg/ml) were used to observe the dose effect of exosomes. After exosomes supplementation, cells were then treated with H_2_O_2_ for 18 h to induce apoptosis and then stained with Annexin-V-propidium iodide double staining (Fig. 6c). When cells were incubated without exosomes, the proportion of apoptotic cells was 25.4% ± 1.23 after H_2_O_2_ treatment. However, the proportion of apoptotic cells was 15.5% ± 1.12 (10 μg/ml) and 12.0% ± 1.31 (20 μg/ml) when cells were incubated with exosomes (Fig. 6d). Therefore, we can infer that supplementation with exosomes is capable of inhibiting apoptosis in CCSMCs.

### Effects of exosomes supplementation on the caspase-3 activity

To explore whether the supplementation of exosomes to culture medium was capable of inhibiting caspase-3 activation in CCSMCs, we investigated caspase-3 activation by western blot analysis. CCSMCs were cultured in the absence or presence of exosomes for 6 h. Different concentrations of exosomes (10 μg/ml, 20 μg/ml) were used to observe the dose effect of exosomes. Cells were then treated with H_2_O_2_ for 18 h to induce apoptosis, and caspase-3 activities were measured in cell lysates (Fig. 6e). As shown in Fig. 6f, the expression of caspase-3 significantly decreased in cells supplemented with exosomes (10 μg/ml, 0.94 ± 0.05; 20 μg/ml, 0.98 ± 0.05) compared to cells cultured without exosomes (1.25 ± 0.07, *p* < 0.01). This indicated that the treatment with exosomes protected CCSMCs from the caspase-3-dependent apoptosis pathway.

### MSC-Exos uptake in vitro and in vivo

**Fig. 7:**
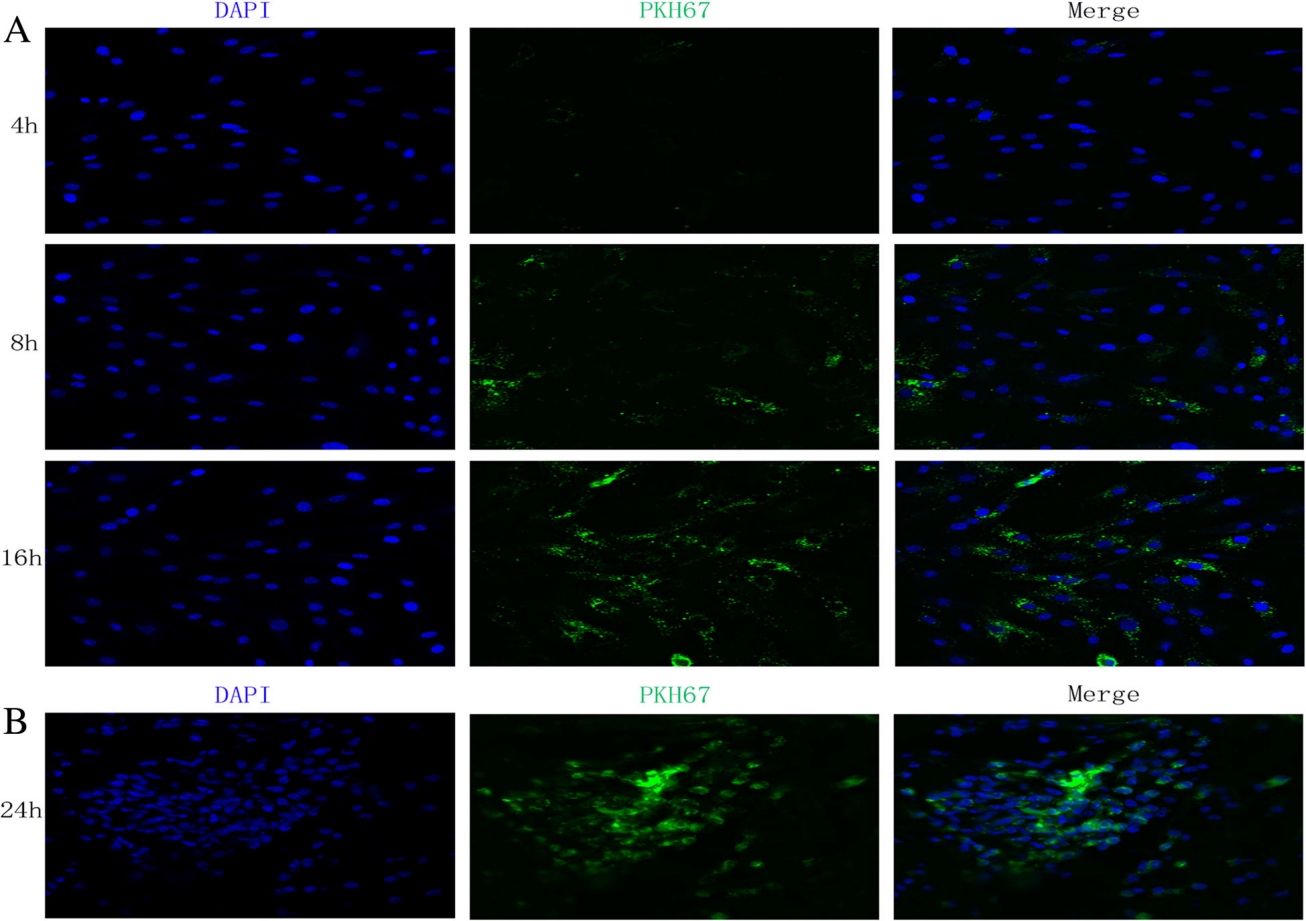
MSC-Exos uptake in vitro and in vivo. **a** The internalization of exosomes into CCSMCs was detected by fluorescence microscopy after CCSMCs were co-cultured with PKH26-labeled exosomes for 4 h, 8 h and 16 h, ×200 amplification. B.PKH26-labeled exosomes were observed by immunofluorescence after injected intracavernous, ×200 amplification

Next, we determined whether MSC-Exos could be internalized into CCSMCs and cavernosum cells. MSC-Exos were labeled by PKH67 and incubated with CCSMCs or injected into the cavernosum. Fluorescence microscopy analysis revealed that the PKH67-labeled exosomes had been transferred to the perinuclear region of CCSMCs (Fig. 7a) and cavernosum cells (Fig. 7b). This result implied that MSC-Exos have the potential to communicate directly with CCSMCs and cavernosum cells and exert their anti-apoptotic effects.

## Discussion

Intracavernous injections of MSCs can enhance the recovery of erectile function in rats with CNI-induced ED. The paracrine mechanisms of MSCs are currently considered to have a major role. Exosomes, which are important bioactive substance vectors secreted by MSCs, have never been investigated as a treatment for CNI-induced ED. In this study, we isolated exosomes from the culture supernatants of MSCs and observed their efficacy in ameliorating CNI-induced ED in rats, as defined by higher ICP/MAP ratios and nNOS expression, higher smooth muscle/collagen ratios, as well as reduced caspase-3 expression. We also demonstrated in vivo and in vitro that MSC-Exos could be internalized into cavernosum cells and CCSMCs, inhibiting the caspase-3 dependent apoptosis pathway.

Exosomes have been demonstrated to be an important mode of cellular communication [20], as they are involved in multiple physiologic and pathologic functions, including proliferation [31], apoptosis [32], inflammation [33], and tissue regeneration [34]. MSC-Exos contain growth factors, signaling lipids, mRNAs, and regulatory miRNAs, which are involved in tissue repair as paracrine mediators. A growing number of studies suggest that MSCs and MSC-Exos yield similar therapeutic benefits in various disease models [23, 35]. It has also been shown that the exosomes released by MSCs can ameliorate diabetes mellitus (DM)-related ED by anti-fibrotic and anti-apoptotic mechanisms and increase endothelial and smooth muscle content [36, 37]. Exosomes-mediated cell-free therapies have many advantages compared with cell therapy, including the following: (i) they have greater stability and are easier to store and manage because exosomes can be stored at − 20 °C for 6 months with no loss of biochemical activity [38], (ii) they do not present a risk of tumor formation, and (iii) exosomes are less immunogenic than cells. Thus, exosomes released by MSCs have the potential to be exploited as a novel alternative to whole-cell therapy.

CCSMCs are the major cells involved in erectile function and dysfunction. Apoptosis of smooth muscles occurs with CNI due to lack of penile innervation. Many studies have suggested that the increased apoptosis of CCSMCs is a common etiology of CNI-induced ED [8, 39]. A previous study found that CNI-induced damage to corporal smooth muscle cells was irreversible [4], and most treatment strategies for CNI-induced ED become ineffective once smooth muscle apoptosis occurs [40]. Wu et al. [4] observed partial spontaneous recovery of the CN at 28 d post-injury by ultrastructural analyses. Therefore, focusing on protecting the corpus cavernosum from apoptosis while the CN regenerates will accelerate the resumption of normal erectile function, and fibrosis would be prevented. In this study, we evaluated the effects of MSC-Exos on the apoptotic pathway in vivo and in vitro. The results revealed that these nanoparticles could be internalized by cavernosum cells and CCSMCs and significantly reduce the rate of apoptosis. Our results suggest that regulation of apoptosis in CCSMCs is a mechanism through which MSC-Exos rescue CNI-induced ED. Thus, the beneficial effects of MSC-Exos in treatment of CNI-induced ED may be mainly attributed to their anti-apoptotic function in CCSMCs. In the present study, we observed that the expression level of caspase-3 did not decrease with the increase in the concentration of exosomes, which was inconsistent with the change of apoptotic rate. Caspases-3 is an important member of the caspase family, which is crucial in the regulation of apoptosis [41, 42]. The results showed that there may be caspase-3 independent apoptotic pathway involved in the anti-apoptotic effect of exosomes in CCSMCs. Further research needs to be performed to determine the exact mechanisms and pathways involved in the anti-apoptotic function of MSC-Exos.

It has been suggested that exosomes can serve as vehicles to transfer large amounts of miRNAs to recipient cells, therefore altering the gene expression and bioactivity of the recipient cells [43]. Many miRNAs have been shown to be strong anti-apoptotic factors, such as miR-21 [44, 45], miR-124 [46], miR-31 [47],and miR-let-7a [48]. The miRNA expression profile of MSCs is associated with a high expression of the miR-21, miR-31, and miR-let7a [49, 50], suggesting that these miRNAs derived from MSCs may play an anti-apoptotic role in recovery from CNI-induced ED.

There are some limitations in the present study. First, the cargo of the exosomes involved in recovery of ED remains to be identified. A large number of related studies have focused on miRNAs, but the exact mechanism remains to be further studied. Second, although we used two different doses of exosomes for our in vitro assays, we only used a single dose of exosomes, 100 μg, for in vivo experiments. Future studies will be aimed at identifying the optimum dose and the times of injection. Moreover, in our study, we focused on the therapeutic effects of exosomes in CNI-induced ED and did not compare them with the non-exosomal fraction of the media, such as exosomes-depleted conditioned medium (CM-Exos). Our object in this study was to investigate whether treatment of CNI with MSC-Exos provides similar functional benefit compared with MSCs treatment, which may suggest a novel cell-free therapy for CNI-induced ED instead of MSC-based cellular therapy. Multistep centrifugation is widely used for the isolation of exosomes since this method can reduce the contamination of protein in exosomes. In contrast, CM-Exos contain a large amount of serum protein, which lead to a lack of effective control between exosomes and CM-Exos. Furuta et al. [51] used serum-free culture to reduce the confounding factors between exosomes and CM-Exos, but not all cells can tolerate serum-free culture. A more rational experimental design is necessary to elucidate the role of exosomes of the paracrine effect of MSCs in future studies.

## Conclusions

MSC-derived exosomes isolated from MSCs culture supernatants by ultracentrifugation could ameliorate CNI-induced ED in rats by inhibiting the apoptosis of CCSMCs, with a similar potency to that observed in the MSCs group. Therefore, this cell-free therapy has a great potential for application in the treatment of CNI-induced ED for replacing cell therapy.

## Abbreviations

CCSMCs: Corpus cavernosum smooth muscle cells
CNI: Cavernous nerve injury
ED: Erectile dysfunction
EV: Extracellular vesicle
FBS: Fetal bovine serum
IC: Intracavernosal
ICP: Intracavernous pressure
MAP: Mean arterial blood pressure
MPG: Major pelvic ganglion
MSCs: Mesenchymal stem cells
MSC-Exos: MSC-derived exosomes
PDE51: Phosphodiesterase type 5 inhibitors
TEM: Transmission electron microscopy
