# Correction: MSC-derived exosomes ameliorate erectile dysfunction by alleviation of corpus cavernosum smooth muscle apoptosis in a rat model of cavernous nerve injury

**DOI:** 10.1186/s13287-022-03190-7

**Published:** 2022-10-24

**Authors:** Xi Ouyang, Xiaoyan Han, Zehong Chen, Jiafeng Fang, Xuna Huang, Hongbo Wei

**Affiliations:** 1 Department of Gastrointestinal Surgery, The Third Affiliated Hospital of Sun Yat-Sen University, Tianhe Road 600, Guangzhou, 510630 China; 2 Central Laboratory, The Third Affiliated Hospital of Sun Yat-Sen University, Tianhe Road 600, Guangzhou, 510630 China

## Correction to: Stem Cell Research & Therapy (2018) 9:246 https://doi.org/10.1186/s13287-018-1003-1

The original article [1] contained an error in Fig. 5 which needs to be corrected. A wrong representative α-SMA immunofluorescence staining image of Sham group was used during assembly of the figure, and caused the image overlap between Sham and MSCs groups in Fig. 5a. The corrected Fig. 5 is presented in this correction.

**Figure:**
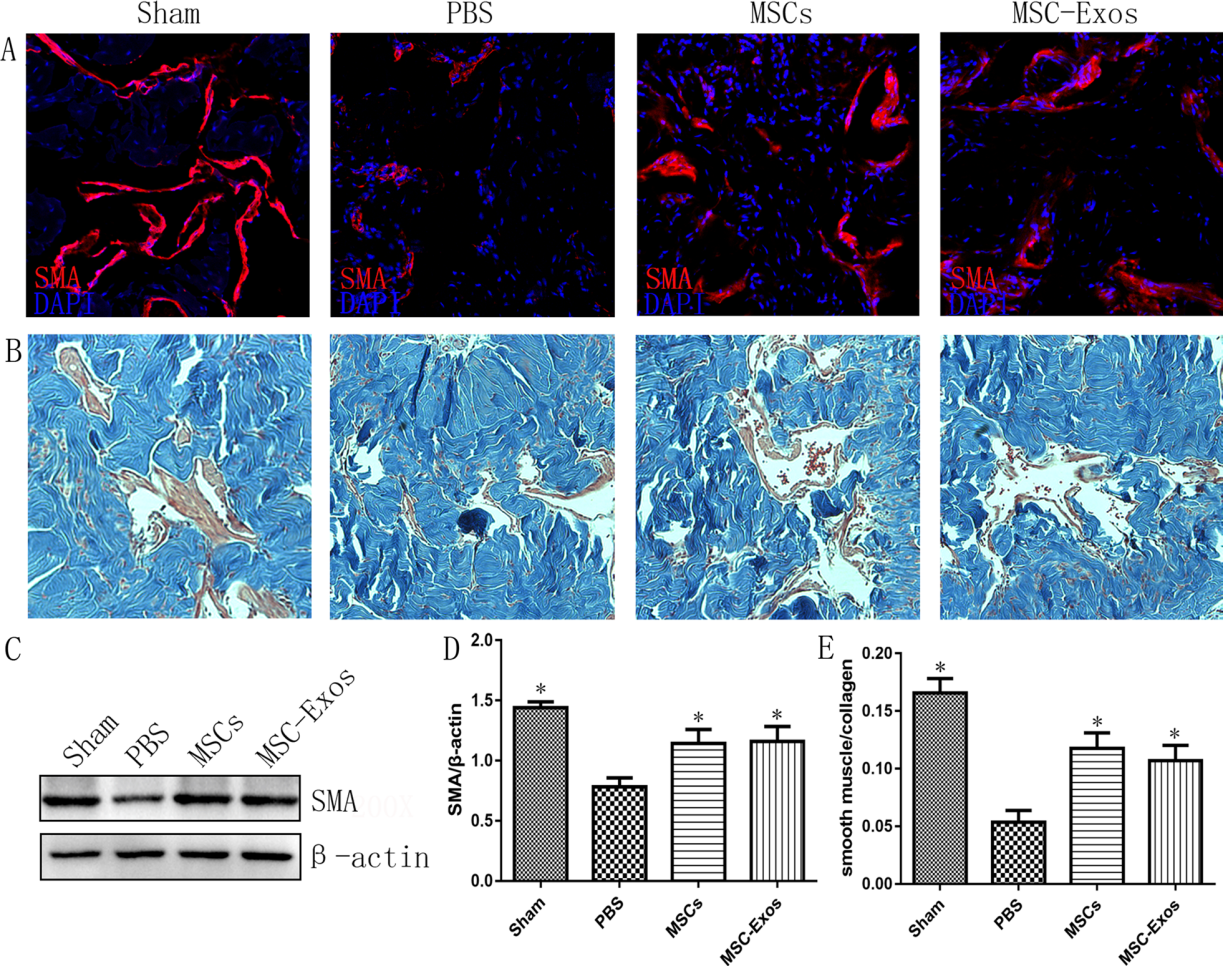
**Fig. 5** Transplantations of MSCs or MSC-Exos increase the smooth muscle contents of the corpus cavernosum. **a** The expressions of SMA in the corpus cavernosum were detected at 4 weeks in each group. Original magnification, ×200 DAPI = 4′,6-diamidino-2-phenylindole. **b** Representative images of Masson trichrome staining of actin and collagen in each group. Smooth muscle and connective tissue in the corpus cavernosum are stained red and blue, respectively. Original magnification,  × 200. **c** Representative images of western blots for SMA cavernosum in each group. **d** Data are presented as the relative density of SMA compared with that of β-actin. The density was determined semiquantitatively using ImageJ. Each bar depicts the means ± standard deviations from n = 8 animals per group. *p < 0.05 compared with the PBS vehicle group. **e** Effect of MSCs or MSC-Exos treatment on the ratio of smooth muscle to collagen in the corpus cavernosum. Bars denote the mean densitometry ratio between smooth muscle content and collagen content per field (± standard error of the mean). *p < 0.05 compared with the PBS vehicle group

In addition, the Fig. 7 legend also needs correction, that the magnification of panel B was mislabeled. The actual magnification of panel B is 100 × .

The correct Figure legend for Fig. 7 is as follows:

Fig. 7 MSC-Exos uptake in vitro and in vivo. A. The internalization of exosomes into CCSMCs was detected by fluorescence microscopy after CCSMCs were co-cultured with PKH26-labeled exosomes for 4 h, 8 h and 16 h, × 200 amplification. B.PKH26-labeled exosomes were observed by immunofluorescence after injected intracavernous, × 100 amplification

The authors state that these mistakes will not affect the result and conclusion of the article.
